# Investigation of Bioactive Compounds Extracted from *Verbena officinalis* and Their Biological Effects in the Extraction by Four Butanol/Ethanol Solvent Combinations

**DOI:** 10.3390/ph18071012

**Published:** 2025-07-07

**Authors:** Dejan Stojković, Nikoleta Đorđevski, Mladen Rajaković, Biljana Filipović, Jelena Božunović, Stefani Bolevich, Gokhan Zengin, Sergey Bolevich, Uroš Gašić, Marina Soković

**Affiliations:** 1 Institute for Biological Research “Siniša Stanković”, National Institute of the Republic of Serbia, University of Belgrade, Bulevar Despota Stefana 142, 11108 Belgrade, Serbia; mladen.rajakovic@ibiss.bg.ac.rs (M.R.); biljana.nikolic@ibiss.bg.ac.rs (B.F.); jelena.boljevic@ibiss.bg.ac.rs (J.B.); uros.gasic@ibiss.bg.ac.rs (U.G.); 2 Institute of Microbiology, Medical Military Academy, Crnotravska 17, 11000 Belgrade, Serbia; nikoleta.djordjevski@gmail.com; 3 Department of Pathologic Physiology, First Moscow State Medical University I.M. Sechenov (Sechenov University), Trubetskaya Street, House 8, Building 2, 119991 Moscow, Russia; alistra555@mail.ru (S.B.); bolevich2011@yandex.ru (S.B.); 4 Department of Biology, Science Faculty, Selcuk University, Campus-Konya, 42250 Konya, Turkey; gokhanzengin@selcuk.edu.tr

**Keywords:** *Verbena officinalis*, LC-HRMS/MS, antioxidant activity, enzyme inhibition, molecular docking, antimicrobial activity

## Abstract

**Background/Objectives**: *Verbena officinalis* L. (common vervain) is a medicinal plant traditionally used and investigated in phytotherapy for its neuroprotective, antioxidant, and anti-inflammatory properties. This study aims to investigate the phytochemical diversity and biological activity of *V. officinalis* extracts prepared with different ratios of butanol and ethanol. **Methods:** Aerial parts of *V. officinalis* were extracted using four solvent systems: 100% butanol (B1), 75:25 (BE7.5), 50:50 (BE5), and 25:75 (BE2.5) butanol:ethanol mixtures. Metabolite profiling was conducted using liquid chromatography–high-resolution tandem mass spectrometry (LC-HRMS/MS). Antioxidant activities were evaluated through six assays: 2,2-diphenyl-1-picrylhydrazyl (DPPH), 2,2′-azino-bis(3-ethylbenzothiazoline-6-sulfonic acid) (ABTS), cupric ion-reducing antioxidant capacity (CUPRAC), ferric-reducing antioxidant power (FRAP), metal-chelating ability (MCA), and the phosphomolybdenum assay (PMA). Enzyme inhibition assays targeted acetylcholinesterase (AChE), butyrylcholinesterase (BChE), tyrosinase, and α-amylase. Antibacterial activity against *Pseudomonas aeruginosa* was tested via microdilution, while dominant phytochemicals were evaluated for binding affinity through molecular docking. **Results:** Seventy-five compounds, including phenolic acids, flavonoids, iridoids, phenylethanoids, and xanthones, were identified. BE5 extract exhibited the highest total phenolic content and strongest antioxidant capacity, while BE2.5 demonstrated the greatest antibacterial and metal-chelating effects. All extracts showed comparable AChE inhibition, with BE5 achieving the strongest tyrosinase and α-amylase inhibition. Docking studies confirmed high binding affinities of luteolin glucuronides to human and bacterial target enzymes. **Conclusions:** Solvent composition markedly influenced the chemical and biological profiles of *V. officinalis* extracts. BE5 and BE2.5 emerged as promising systems for obtaining bioactive fractions with therapeutic potential.

## 1. Introduction

*Verbena officinalis* L. (common vervain, Verbenaceae) is a highly valuated medicinal plant native to the Old World and introduced to Australia and South and North America [1]. It is a perennial herbaceous plant that prefers sunny locations with dry soils and can be found as a ruderal species near roads, on stone rubble, in fields, and wastelands [2]. This widely distributed species is used in folk medicine of various countries and in traditional Chinese medicine for the treatment of many ailments: rheumatism, anxiety, depression, insomnia, bronchitis, dysmenorrhea, amenorrhea, throat swelling, abscesses, edema, jaundice, malaria, and liver and gallbladder diseases [3]. Ethnopharmacological records of ancient uses of this plant are well documented and confirm that it was considered a “sacred herb” in European folk medicine [4]. *Verbenae herba*, a raw material consisting of the aerial parts, is included in both the Chinese Pharmacopoeia and the European Pharmacopoeia [3].

The various biological effects of the extracts and essential oils are attributed to the very rich and diverse chemical profile of the *V. officinalis* herb. The major groups of pharmacologically valuable compounds are iridoids, phenylethanoid glycosides, phenolic acids, and flavonoids [3,5,6]. Essential oil is dominated by terpenoids (monoterpenoids, diterpenoids, triterpenoids, sesquiterpenoids, and sterols) [3,4]. *Verbena officinalis* has been shown to contain bioactive pentacyclic triterpenoids, including ursolic acid derivates and novel compounds such as officinalisoids A-D, which exhibit anti-inflammatory and antitumor properties [5,7,8,9,10]. Iridoids, specialized metabolites with a broad spectrum of pharmacological activities, are characteristic components of this plant. The most abundant iridoids in *V. officinalis* are verbenalin, hastatoside, and aucubin. Iridoids in *V. officinalis* are recognized as sleep-promoting components (hastatoside and verbenalin) [11], and as components that promote host immune homeostasis and protect against coronavirus pneumonia (verbenalin) [12]. Among flavonoids, quercetin, luteolin, kaempferol, scutellarein, and pedalitin were identified as dominant compounds [13,14]. Luteolin-7-diglucuronide, a major flavonoid extracted from *V. officinalis* and *Perilla frutescens*, attenuates liver fibrosis in mice by inhibiting tyrosine phosphatase 1B (PTP1B) activity [15]. The most characteristic phenylethanoid glycosides are verbascoside, isoverbascoside, and eukovoside. Phenolic acids identified are chlorogenic, ferulic, protocatechuic, rosmarinic, and quinic acid derivates.

The major constituents identified in *V. officinalis* extracts are verbenalin and verbascoside. According to the European Pharmacopeia, the content of verbenalin as the quality determining compound in the standardized *Verbenae herba* should not be less than 1.5% dry weight. Phytochemical composition of *V. officinalis* can be influenced by both endogenous and environmental factors (the geographical origin, plant age, the plant part analyzed, harvesting time, etc.), as well as by extraction methods and different solvents [4,16].

Bioactive compounds in *V. officinalis* are recognized as anti-Alzheimer’s disease agents [6].

The aim of this study was to determine phytochemical profile and in vitro biological potential of *V. officinalis* extracts by antioxidant and enzyme inhibition assays. In addition, the targeted regulation of acetylcholinesterase, butyrylcholinesterase, pancreatic alpha amylase, tyrosinase, and selected enzymes representing virulence factors in *P. aeruginosa* by six of the most abundant compounds in the extract iridoids (verbenalin and hastatoside), flavonoids (luteolin 7-*O*-(2″-glucuronyl)-glucuronide and luteolin-7-*O*-glucuronide), and phenylethanoid glycosides (verbascoide and martynoside) was validated through molecular docking studies.

## 2. Results and Discussion

### 2.1. Phytochemical Mapping of Common Vervain

The yields of extracts, calculated as percentage of dry weight, were as follows: B1—9.38%; BE7.5—7.56%; BE5—11.23%; and BE2.5—6.83%. The content of total phenolics and flavonoids in the aerial parts of four *Verbena officinalis* extracts is summarized in Table 1. Generally, the highest amount of total phenolics and total flavonoids was noted in the BE5 extract, while the lowest content was discovered in the BE7.5 extract. Butanol fractions of *V. officinalis* have been previously described as rich in secondary metabolites, especially phenolic compounds [17]. The results presented in Table 1 indicate that the combination of two solvents in a 50:50 ratio (BE5) is the most efficient method for the isolation of phenolic compounds.

Chemical analysis of bioactive compounds extracted from *Verbena officinalis*, conducted using the high-resolution LC/MS technique, led to the identification of 75 compounds. Metabolite identification was achieved through the study of the exact mass of the compounds in a full-scan experiment, as well as MS^2^ fragmentation at high resolution. The structures of the compounds were proposed based on a comprehensive review of the literature on *Verbena* metabolites, where available. Base peak chromatograms of all four different extracts are depicted in Figure S1, and there it can be seen that the most abundant peaks are hastatoside and verbenalin, whose molecular ions in our experiment appear as formic acid adducts. Table S1 lists the identified compounds, together with retention times and key mass spectrometry attributes (exact mass and MS^2^ fragmentation), divided into several groups according to their basic chemical structures: benzoic acid derivatives (12 compounds), cinnamic acid derivatives (10 compounds), iridoid glycosides (8 compounds), phenylethanoids (8 compounds), flavonoid C-glycosides (3 compounds), flavonoid O-glycosides (14 compounds), flavonoid aglycones (14 compounds), and xanthones (6 compounds). Notably, the majority of detected metabolites belong to the groups of iridoid glycosides, followed by flavonoid glycosides and aglycones. These findings are in line with previous reports but also provide additional insight into the chemical complexity of this species.

Among the most relevant identified compounds were rosmarinic acid, verbascoside, verbenalin, and hastatoside, which are consistently reported as key constituents of *V. officinalis* in earlier studies [13,17,18]. The presence of rosmarinic acid, a major antioxidant phenolic compound, supports findings by Rehecho et al. (2011) [13], who associated this molecule with the high antioxidant capacity of the plant. Likewise, verbenalin and hastatoside, two bioactive iridoid glycosides with reported anti-inflammatory, sedative, and neuroprotective properties [6,19], were also confirmed in our profile.

In addition to known compounds, this analysis identified a wide range of flavonoid derivatives, including apigenin, luteolin, quercetin, and isorhamnetin, both in aglycone and glycosylated forms. These flavonoids have previously been associated with anti-inflammatory and neuroprotective activity [6]. The detection of flavonoid C-glycosides (e.g., apigenin 6,8-di-C-hexoside) and acetylated/rhamnosylated forms (e.g., myricetin 3-O-(acetyl)-rhamnoside, quercetin 3-O-(acetyl)-rhamnoside) suggests a complex and diverse flavonoid metabolism that has not been equally emphasized in earlier studies.

Interestingly, several xanthone derivatives, including trihydroxy- and dihydroxy-methoxyxanthones, were identified. These compounds are less frequently reported in *V. officinalis*, and their presence may represent chemotaxonomic markers or be indicative of environmental influences on secondary metabolite biosynthesis. Some compounds reported by El-Wakil et al. (2022) [17], such as specific sesquiterpenes or non-polar components, were not detected in our LC-MS profile, likely due to methodological differences in detection sensitivity or polarity of the extraction solvents.

**Figure 1:**
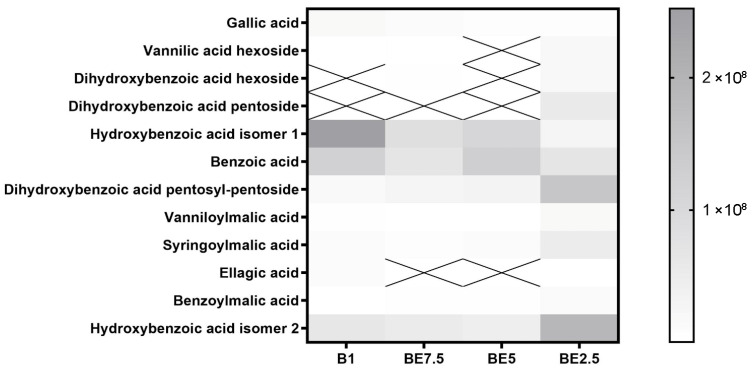
Benzoic acid derivatives in *Verbena officinalis* extracts obtained using varying ratios of butanol and ethanol (B1, BE7.5, BE5, and BE2.5). B1—100% butanol; BE7.5—butanol:ethanol (75%:25%); BE5—butanol:ethanol (50%:50%); BE2.5—butanol:ethanol (25%:75%). X on the heatmap presents that compound was not detected in particular sample.

**Figure 2:**
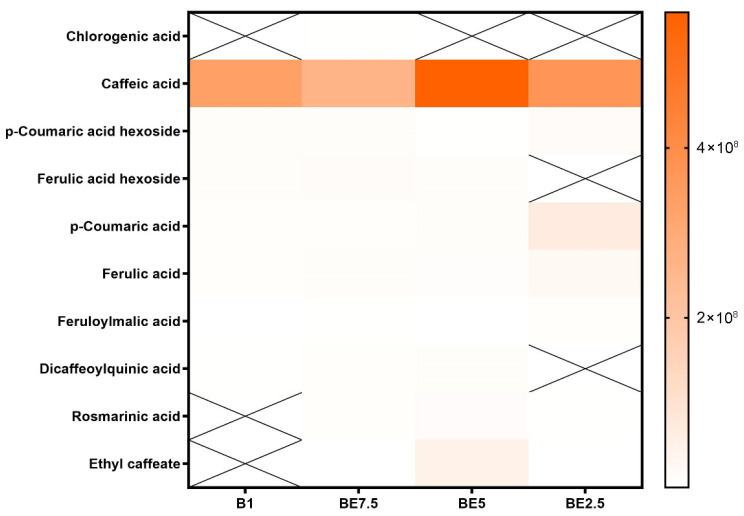
Distribution of cinnamic acid derivatives in *V. officinalis* extracts obtained with different butanol–ethanol solvent ratios: B1 (100% butanol), BE7.5 (75:25), BE5 (50:50), and BE2.5 (25:75). “X” indicates absence of the compound in the corresponding extract.

**Figure 3:**
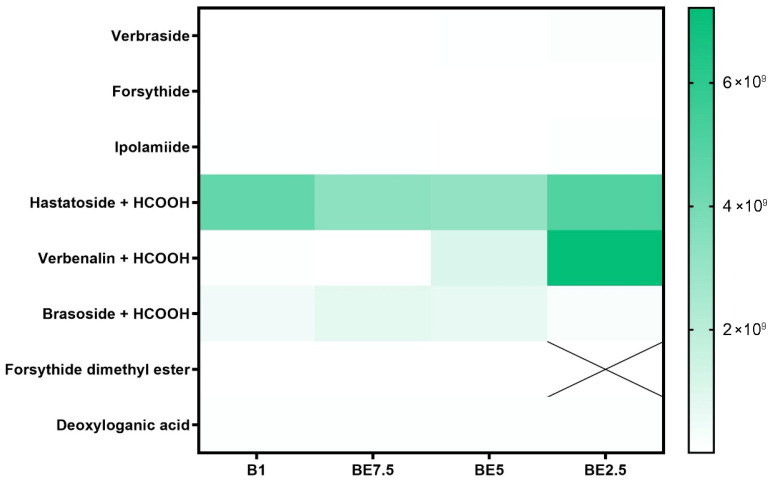
Heatmap showing the presence of iridoid glycosides in *V. officinalis* extracts prepared using increasing proportions of ethanol in butanol: B1 (pure butanol), BE7.5 (75% butanol), BE5 (50%:50%), and BE2.5 (25% butanol). Compounds marked with “X” were not detected in the respective sample.

**Figure 4:**
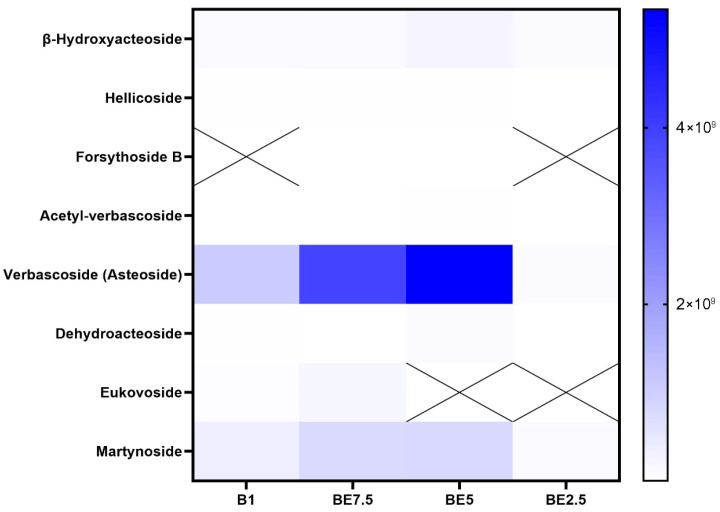
Heatmap visualization of phenylethanoids detection across *V. officinalis* extracts prepared with varying butanol:ethanol ratios: B1 (100% butanol), BE7.5 (75:25), BE5 (50:50), and BE2.5 (25:75). Absence of a compound is represented by “X”.

**Figure 5:**
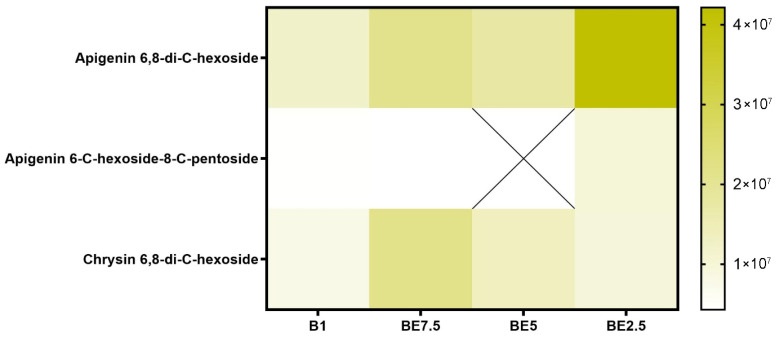
Comparative occurrence of flavonoid *C*-glycosides in *V. officinalis* extracts using four solvent systems: B1 (100% butanol), BE7.5 (75% butanol), BE5 (1:1), and BE2.5 (25% butanol). “X” indicates non-detection in the corresponding extract.

**Figure 6:**
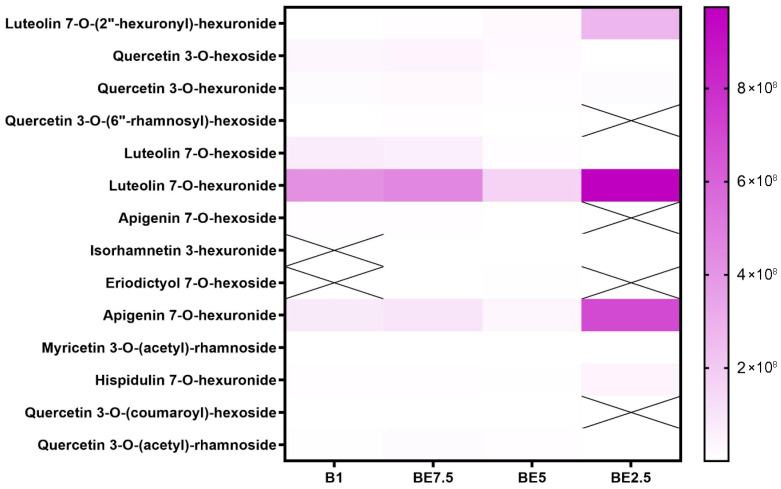
Detection heatmap of flavonoid *O*-glycosides in *V. officinalis* extracts obtained with B1 (butanol), BE7.5 (75% butanol), BE5 (50%), and BE2.5 (25%). Cells marked with “X” represent samples in which the compound was not detected.

**Figure 7:**
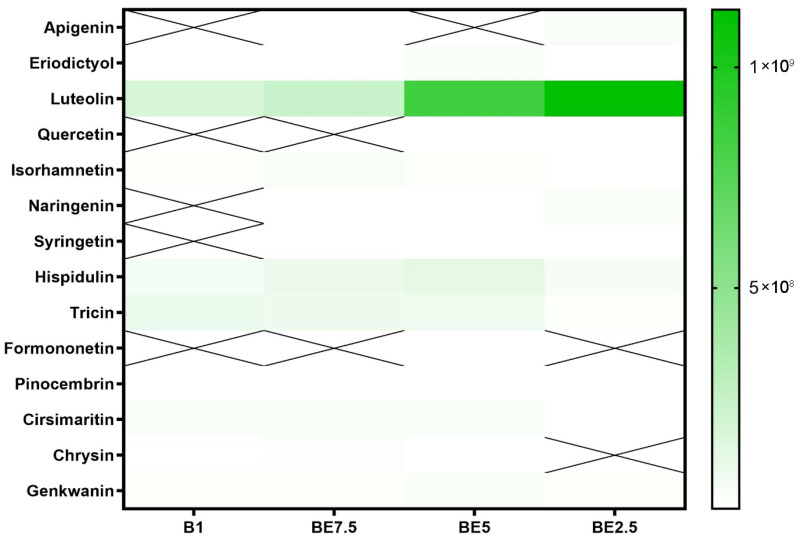
Heatmap illustrating the distribution of flavonoid aglycones in *V. officinalis* extracts produced with different butanol:ethanol ratios: B1 (100% butanol), BE7.5 (75:25), BE5 (50:50), and BE2.5 (25:75). An “X” signifies the compound was not detected in that specific sample.

**Figure 8:**
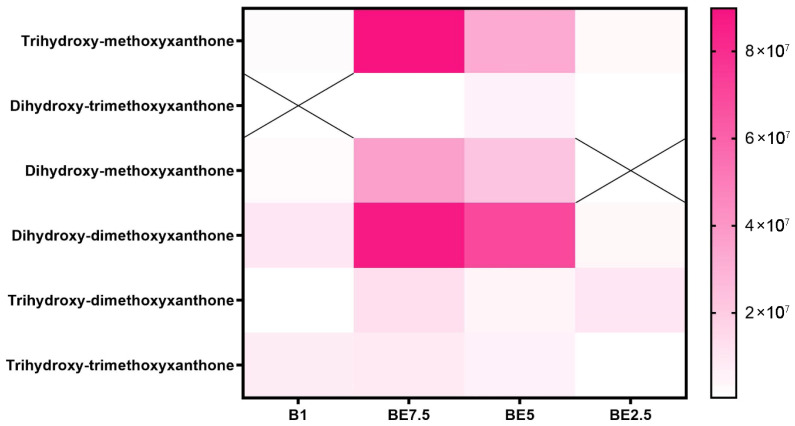
Overview of detected xanthone compounds in *V. officinalis* extracts prepared using various butanol and ethanol mixtures—B1 (pure butanol), BE7.5 (75% butanol), BE5 (equal ratio), and BE2.5 (25% butanol). The symbol “X” indicates the absence of a compound in the corresponding extract.

A set of heatmaps (Figure 1, Figure 2, Figure 3, Figure 4, Figure 5, Figure 6, Figure 7 and Figure 8) was created to show the distribution of individual compounds across four solvent systems of increasing polarity, namely B1 (100% butanol), BE7.5 (75:25 butanol:ethanol), BE5 (50:50), and BE2.5 (25:75), in order to thoroughly assess the phytochemical diversity and extraction efficiency of *V. officinalis*.

Different extraction patterns influenced by solvent polarity are shown in the heatmap of benzoic acid derivatives across four *V. officinalis* extracts (Figure 1). The most abundant and consistent compounds among those found are benzoic acid and hydroxybenzoic acid isomer 1, dihydroxybenzoic acid pentosyl-pentoside, especially in B1 and BE5 samples. This suggests that these relatively simple phenolic acids are easily extracted across a range of polarities, with a slight preference for mid-polar systems. Good solubility in ethanol-rich solutions is indicated by the moderate and uniform distribution of hydroxybenzoic acid isomer 2, and dihydroxybenzoic acid pentoside, vanillic acid hexoside, dihydroxybenzoic acid hexoside. Their increased polarity and affinity for ethanol-enriched solvents are reflected in their more pronounced extraction in BE2.5. All extracts include very low intensities of compounds, such as gallic acid, benzoylmalic acid, syringoylmalic acid, and vanilloylmalic acid, with no clear solvent preference. BE2.5 demonstrates the broadest and most balanced extraction capacity for benzoic acid derivatives, recovering both free acids and more polar glycosylated forms. These findings highlight the importance of tuning solvent polarity to target specific subgroups within the phenolic acid class and underscore the potential of mixed alcohol systems for comprehensive phytochemical recovery.

Caffeic acid is found in all four extracts and has the maximum intensity in BE5, followed by BE2.5 and B1, with the lowest signal in BE7.5, according to the heatmap of cinnamic acid derivatives (Figure 2). This distinct polarity-dependent pattern shows that mid-ethanol solvent systems, especially the 50:50 butanol:ethanol mixture (BE5), which offers the highest extraction yield, are the most effective for extracting caffeic acid, a moderately polar hydroxycinnamic acid. The relevance of caffeic acid as a major phenolic element in extracts is shown by its widespread occurrence across samples. The majority of other derivatives of cinnamic acid, on the other hand, show very little or no detection, indicating either low natural abundance or poor solubility under the extraction conditions used. Once more demonstrating the significance of polar solvents, p-coumaric acid and ferulic acid exhibit very low intensity, with modest signals in BE2.5 and BE5. The results provide compelling evidence that the best solvent for obtaining hydroxycinnamic acids from *V. officinalis* is BE5 (50:50 butanol:ethanol), especially caffeic acid, which seems to be the most prevalent component.

Iridoid glycosides’ heatmap (Figure 3) shows a distinct polarity-dependent extraction pattern, with BE2.5 and, to a lesser extent, BE5 showing the most effective recovery. Out of all the chemicals listed, hastatoside + HCOOH has the largest and most stable abundance in all four extracts, peaking in BE2.5. Its high natural content and generally advantageous solubility in a range of polarities are highlighted by its extensive presence across extraction techniques. The second most common compound is verbenalin + HCOOH, whose extraction gets better over time from B1 to BE2.5. Its polarity and the beneficial effects of ethanol on solubilization and matrix permeability are evident in this trend. The appropriateness of mid-ethanol concentrations for the extraction of glycosidic iridoids is further supported by the low to moderate quantities of brasoside + HCOOH that are present in all extracts, with a minor enrichment in BE7.5 and BE5. The examined sample also contains other iridoid glycosides, including verbraside, forsythide, and ipolamiide. The fact that forsythide dimethyl ester is completely undetectable as well (designated with a “X” in BE2.5) highlights the solvents selectivity. The findings show that the best extracts for obtaining the main iridoid glycosides from *V. officinalis* are those that are ethanol-rich, especially BE2.5. This is especially true for extracts that contain formate derivatives, such as verbenalin and hastatoside.

Verbascoside, often referred to as acteoside, is clearly dominant in all examined extracts, according to the phenylethanoids heatmap (Figure 4), with BE5 showing the highest intensity, followed by BE7.5 and B1. This pattern makes it abundantly evident that a 50:50 butanol:ethanol mixture (BE5) provides the best polarity for verbascoside extraction. Verbascoside is a molecule that requires a moderately polar environment for effective solubilization due to its numerous hydroxyl groups and glycosidic linkages. Verbascoside’s constant high concentration highlights both its significance as a chemotaxonomic and pharmacologically active marker and its richness in the plant material. Martynoside appears in all extracts, suggesting moderate solubility. Other phenylethanoids, including dehydroacteoside, acetyl-verbascoside, hellicoside, and *β*-hydroxyacteoside, are found at much lower concentrations, with weaker signals in extracts. Despite having structural similarities to verbascoside, some derivatives might be less abundant in nature or might be harder to extract under the tested circumstances. They may need specific extraction techniques or be minor ingredients, as indicated by their low intensities in all extracts. Forsythoside B and eukovoside exhibit intermittent detection. Because of their low native abundance or poor solubility in the chosen solvent systems, eukovoside and forsythoside B are only found in a few extracts or not at all. Verbascoside is the most abundant and extractable phenylethanoid in vervain extracts, according to the data, and the BE5 extract performs noticeably better than the others in terms of recovery efficiency.

The most prevalent identified molecule, according to the flavonoid *C*-glycoside heatmap (Figure 5), is apigenin 6,8-di-*C*-hexoside, which is present in all four extracts. The BE2.5 extract has the largest abundance, suggesting a strong preference for extraction under conditions that are high in ethanol. This molecule is relatively polar due to its double glycosylation via C-C bonds, and its extraction is improved with increasing solvent polarity. This compound’s great natural abundance in the plant matrix is further supported by the fact that it is present in all extracts, although to differing degrees. Additionally, chrysin 6,8-di-*C*-hexoside is consistently found in all extracts. The uneven distribution of apigenin 6-*C*-hexoside-8-*C*-pentoside may indicate reduced abundance, variations in stability, or selective extraction based on the delicate relationships between solvent polarity and glycoside composition. All things considered, this heatmap shows that the most effective extracts for apigenin *C*-glycosides are those that are high in ethanol, especially BE2.5.

Luteolin 7-*O*-hexuronide is the most prevalent and reliably extracted flavonoid of the components listed; it is especially prominent in BE2.5, where it reaches its maximum intensity (Figure 6). Additionally, it is found in BE5 and is well-represented in BE7.5 and B1, suggesting that it is relatively soluble in a variety of polarities, although obviously preferring more polar ones. The strong extraction of luteolin 7-*O*-hexoside and apigenin 7-*O*-hexuronide in BE2.5 further supports the idea that ethanol-rich environments are the best conditions for the recovery of these flavonoids. However, certain extracts do not contain compounds such as isorhamnetin 3-hexuronide, eriodictyol 7-*O*-hexoside, and quercetin 3-*O*-(6″-rhamnosyl)-hexoside. Furthermore, only at low intensities are more complicated acylated forms found, such as myricetin 3-*O*-(acetyl)-rhamnoside, quercetin 3-*O*-(coumaroyl)-hexoside, and quercetin 3-*O*-(acetyl)-rhamnoside. This heatmap provides compelling evidence that ethanol-dominant solvents are the most effective for extracting flavonoid *O*-glycosides, especially glucuronidated versions of luteolin and apigenin. The best extraction method for these substances turns out to be BE2.5.

Luteolin is the most prevalent compound in the class of flavonoid aglycones, according to the heatmap depicting them (Figure 7). It can be found in smaller quantities in BE7.5 and B1, but it is most prominent in BE5, with much greater intensity in BE2.5. Given the comparatively low polarity of flavonoid aglycones, this pattern is a little surprising, but it implies that luteolin may still prefer ethanol-rich solvents even though it is an aglycone because it contains several hydroxyl groups, which provide intermediate polarity. Given its great abundance, it is evident that *V. officinalis* contains a significant amount of this bioactive flavone, which has cytoprotective and anti-inflammatory properties. The moderate representation of other aglycones, such as hispidulin, tricin, and cirsimaritin, which are mostly detectable in BE5 and BE7.5, suggests that ethanol-containing solvents are also useful for their extraction. These substances are structurally similar to luteolin, but their substitution patterns are different, which could explain why they are not as soluble.

Apigenin, quercetin, naringenin, syringetin, formononetin, and chrysin are among the other aglycones that are either nonexistent or just occasionally seen in extracts. Low natural concentrations in the plant material or specific solubility profiles that the studied extraction techniques are unable to adequately handle could be the cause of their limited presence. It is interesting to note that formononetin was found in BE5, indicating that this molecule may be extractable in conditions of balanced polarity, but it may only be found in trace amounts in this plant. When creating bioactive extracts from this species, the extraction pattern demonstrates the importance of using hydroalcoholic solvents to maximize aglycone recovery.

Across the four examined solvent systems, the xanthone distribution heatmap (Figure 8) shows a profile of these phenolic chemicals that is extract-specific but generally balanced. The most prevalent molecules are trihydroxy-methoxyxanthone and dihydroxy-dimethoxyxanthone, which are especially abundant in the BE7.5 extract. BE5 has a considerable presence of these compounds, and B1 and BE2.5 have modest detection of them. This pattern suggests that these xanthones are best extracted using a moderately polar solvent composition (75:25 butanol:ethanol), most likely because of their amphipathic nature brought on by both hydroxyl and methoxy substitutions.

According to these results, BE7.5 provides the most effective and varied xanthone extraction, especially for those with several methoxy and hydroxyl groups that provide intermediate polarity. The information highlights the significance of adjusting solvent polarity to maximize recovery and reflects the structurally dependent solubility of xanthones. The antioxidant, anti-inflammatory, and antibacterial qualities of these chemicals make them noteworthy, which emphasizes the importance of maximizing their extraction from *V. officinalis*.

### 2.2. Biological Activities of the Extracts

**Figure 9:**
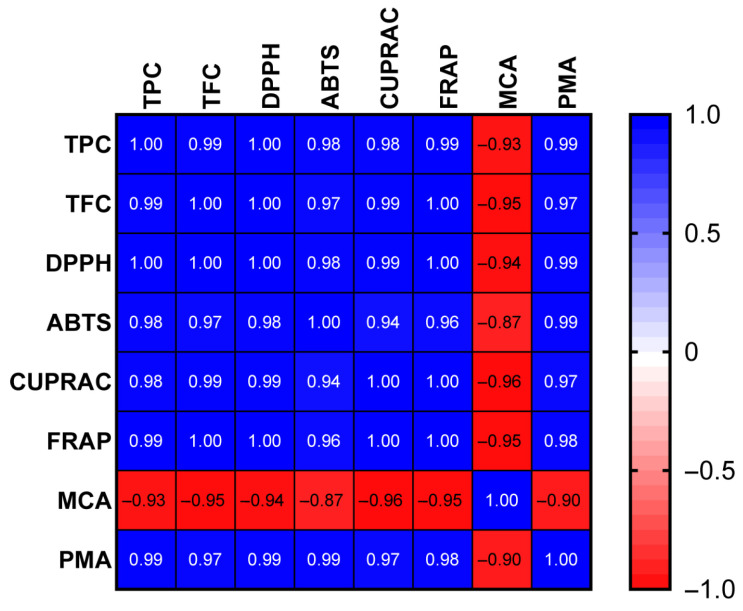
Correlation value between total phenolics/flavonoids of extracts and antioxidant abilities (R^2^).

Table 2 presents the antioxidant activity of four different extracts (B1, BE7.5, BE5, and BE2.5), as assessed using six standard assays: DPPH, ABTS, CUPRAC, FRAP, MCA, and PMA. Among all samples, BE5 exhibited the highest antioxidant activity in the DPPH (181.03 mg TE/g), ABTS (178.50 mg TE/g), CUPRAC (287.86 mg TE/g), and FRAP (170.99 mg TE/g) assays. These results are consistent with its presumed high phenolic content and suggest a strong capacity for radical scavenging and reducing power. Notably, BE5 also recorded the highest activity in the PMA assay (3.62 mmol TE/g), reflecting significant phosphomolybdenum-reducing potential. This may be attributed to the presence of verbascoside, a compound well-documented for its antioxidative properties [20]. In contrast, the BE2.5 and B1 extracts exhibited the best performance in the MCA assay. Iridoid and flavonoid glucosides—particularly verbenalin, hastatoside, and apigenin glucoside—are most abundant in the BE2.5 extract, which is characterized by pronounced metal-chelating activity. To assess the correlation between bioactive compounds and antioxidant properties, we conducted a Pearson correlation analysis. The findings, depicted in Figure 9, indicate that, except for metal-chelating capacity, all antioxidant properties exhibited a strong correlation with the total phenolics/flavonoids content (R^2^ > 0.8). This observation highlighted that phenolic compounds were the primary contributors to the antioxidant properties of *V. officinalis.*

Among the samples tested, all extracts demonstrated comparable AChE inhibitory activity, with values ranging narrowly from 5.10 to 5.17 mg GALAE/g (Table 3), suggesting similar capacities to inhibit enzymes involved in neurodegenerative processes, such as Alzheimer’s disease [6]. BE5 demonstrated the strongest tyrosinase inhibitory activity (102.33 mg KAE/g), surpassing all other extracts. This highlights its potential for applications in managing hyperpigmentation and oxidative-stress-related dermatological conditions. In contrast, BE2.5 exhibited the lowest α-amylase inhibitory activity (0.29 mg ACAE/g), while BE5 again showed the highest (0.59 mg ACAE/g), suggesting a broader spectrum of bioactivity that may be linked to its phytochemical richness.

Taken together, the results demonstrate a relation between the phytochemical composition of the extracts, their antioxidant potential, and their enzyme inhibitory capacities, with BE5 standing out as the most promising candidate for further pharmacological exploration.

Table 4 displays the minimum bactericidal concentrations (MBC) and minimum inhibitory concentrations (MIC) of four extracts (B1, BE7.5, BE5, and BE2.5) that were evaluated against *Pseudomonas aeruginosa*. With different extraction solvent compositions, a distinct trend in antibacterial potency can be seen. Among the extracts, the BE2.5 extract (25% butanol: 75% ethanol) exhibited the lowest MIC (0.25 mg/mL) and MBC (0.50 mg/mL) values, indicating the strongest antibacterial activity. This implies that compounds active against *P. aeruginosa* are better extracted from solvent mixtures with a higher ethanol content. The antimicrobial activity followed the order: BE2.5 > BE5 > BE7.5 = B1 Nevertheless, ampicillin had lower MIC and MBC values (0.003 and 0.005 mg/mL, respectively) and was far more potent than any of the extracts. According to these findings, the extract’s antibacterial activity is highly influenced by the solvent’s polarity, and BE2.5 stands out as the most promising option for more research against *P. aeruginosa*. A previous study reported the antimicrobial activity of various fractions of *Verbena carolina* against *Staphylococcus aureus*, *Enterococcus faecalis*, *Escherichia coli*, *Salmonella typhi*, *Candida albicans*, *Trichophyton mentagrophytes*, and *T. rubrum*. The major compounds identified in these fractions include hispidulin, verbenaline, hastatoside, and verbascoside. Our findings are consistent with these previously reported results on *V. carolina* [21].

### 2.3. Molecular Modeling

**Figure 10:**
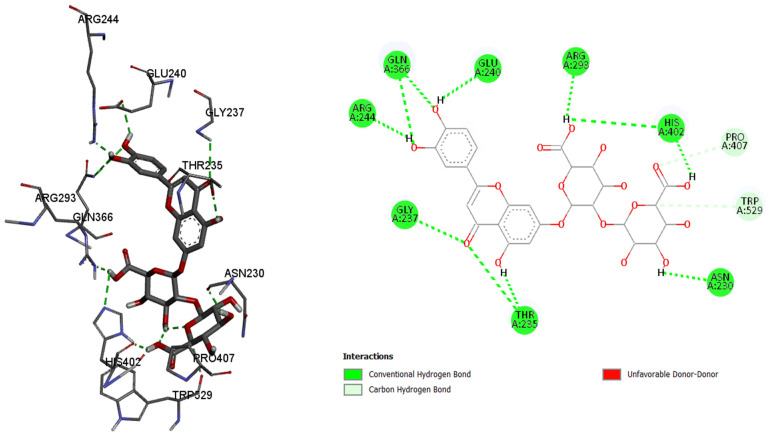
Acetylcholinesterase, amino acids in binding site of the enzyme, 2D plot of the amino acid that correspond to the ligand.

**Figure 11:**
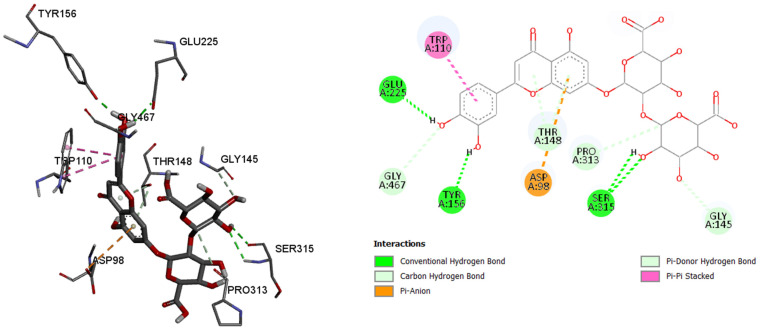
Butyrylcholinesterase, amino acids in binding site of the enzyme; 2D plot of the amino acid that correspond to the ligand.

**Figure 12:**
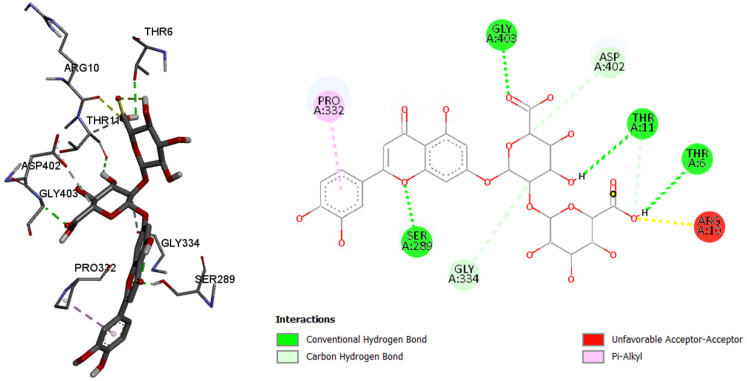
Alpha amylase, amino acids in binding site of the enzyme; 2D plot of the amino acid that correspond to the ligand.

**Figure 13:**
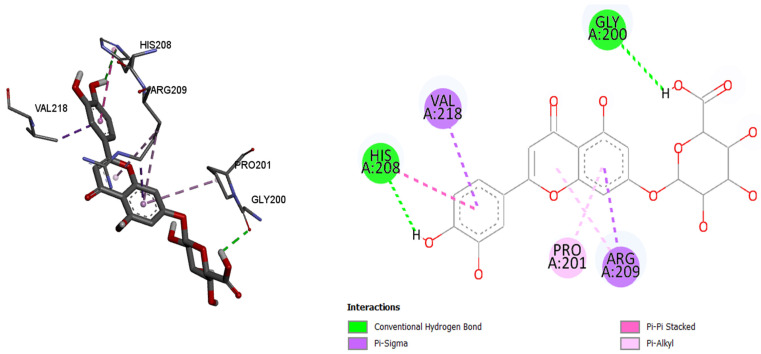
Tyrosinase, amino acids in binding site of the enzyme, 2D plot of the amino acid that correspond to the ligand.

**Figure 14:**
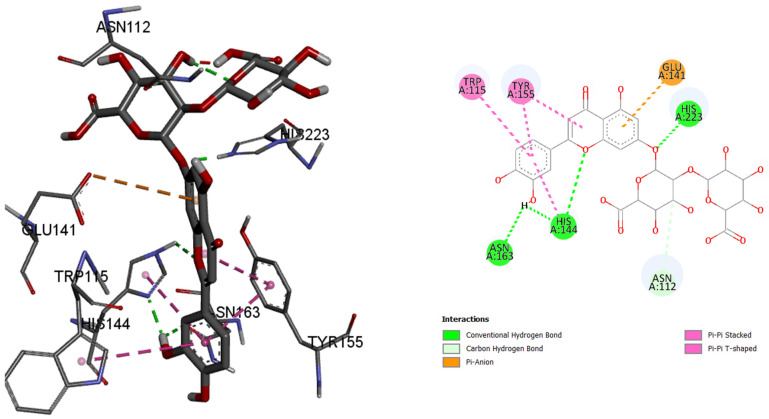
LasB (Elastase B, PDB:8CC4), amino acids in binding site of the enzyme, 2D plot of the amino acid that correspond to the ligand.

**Figure 15:**
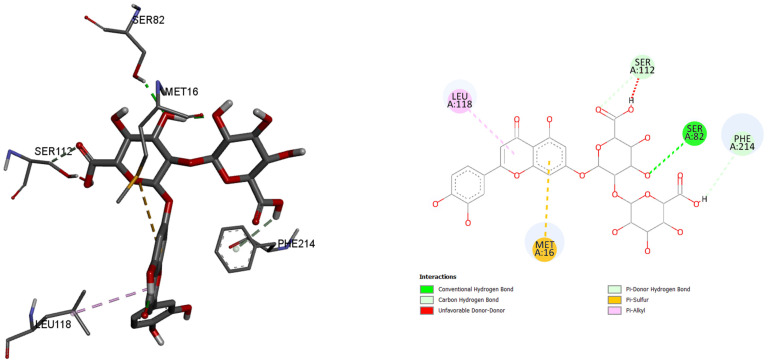
LipA (Lipase A), PDB:1EX9, amino acids in binding site of the enzyme, 2D plot of the amino acid that correspond to the ligand.

Since the most intense peaks were for the compounds hastatoside, luteolin 7-*O*-(2″-glucuronyl)-glucuronide, luteolin-7-*O*-glucuronide, martynoside, verbascoside, and verbenalin, we have carried out further investigation of these compounds and their interactions with enzymes included in anti-enzymatic assays and selected enzymes representing virulence factors in *P. aeruginosa*. Table 5, Table 6, Table 7, Table 8, Table 9 and Table 10 show the docking scores (binding energy) values of the chosen prevalent compounds against each of the six enzymes and the co-crystalized ligands in the active site of enzyme from PDB for each enzyme used as the control molecule. Additionally, the interactions of specific protein–ligand complexes were thoroughly investigated as shown in Figure 10, Figure 11, Figure 12, Figure 13, Figure 14 and Figure 15.

Among the six tested compounds, luteolin 7-*O*-(2″-glucuronyl)-glucuronide shows best binding affinity to the active site of acetylcholinesterase with the binding energy of −10.1 kcal/mol, forming ten hydrogen bonds with the amino acids in the active site. Compared to the control molecule, 1-benzyl-4-[(5,6-dimethoxy-1-indanon-2-yl)methyl]piperidine with the binding energy of −11.4 kcal/mol, luteolin 7-*O*-(2″-glucuronyl)-glucuronide shows less effectiveness towards binding to the active site of acetylcholinesterase.

By analyzing the binding affinity to the active site of the butyrylcholinesterase, the same compound, luteolin 7-*O*-(2″-glucuronyl)-glucuronide, showed the best binding affinity (−10.3 kcal/mol) as in the case of the acetylcholinesterase. Conventional hydrogen bonds are formed between the 7-*O*-(2″-glucuronyl)-glucuronide and the amino acids, namely 225Glu, 156Tyr, and 315Ser, while the π–anion, π–π stacked, π–donor hydrogen, and carbon hydrogen bonds are formed between the rest of the six binding amino acids, and their residues. By comparing the binding affinity of the co-crystalized control molecule (2-{1-[4-(12-Amino-3-chloro-6,7,10,11-tetrahydro-7,11-methanocycloocta[b]quinolin-9-yl)butyl]-1*H*-1,2,3-triazol-4-yl}-*N*-[4-hydroxy-3-methoxybenzyl]acetamide), and the molecule with the best binding affinity among six tested molecules, the control molecule showed slightly better binding affinity (−10.7 kcal/mol).

Like in the other two previously tested enzymes, in this case, luteolin 7-*O*-(2″-glucuronyl)-glucuronide showed the best binding affinity among the six tested compounds. By comparing with the control molecule, 3,5,7-trihydroxy-2-(3,4,5-trihydroxyphenyl)-4*H*-chromen-4-one, 7-*O*-(2″-glucuronyl)-glucuronide showed better binding affinity to the pancreatic alpha amylase, with the binding energy of −9.5 kcal/mol (comparing to the control molecule binding energy of −7.8 kacl/mol), binding with the six amino acids and their residues in the active site of enzyme via conventional and carbon hydrogen, as well as other bonds.

As in the case of tyrosinase, luteolin-7-*O*-glucuronide showed the best binding affinity (binding energy of −7.9 kcal/mol) by comparing it with the other tested molecules. Luteolin-7-*O*-glucuronide bind to the active site of tyrosinase via conventional hydrogen bonds (208His and 200His), π–sigma, π–π stacked, and π–alkyl bonds (218Val, 201Pro, and 209Arg). Luteolin-7-*O*-glucuronide showed better bind affinity compared to the control compound kojic acid (binding energy −5.4 kcal/mol).

In the case of enzyme elastase B from *P. aeruginosa*, luteolin 7-*O*-(2″-glucuronyl)-glucuronide had best binding affinity with the binding energy of −9.6 kcal/mol, making this ligand a better ‘binder’ to the active site of the enzyme instead of the co-crystalized control molecule. Luteolin 7-*O*-(2″-glucuronyl)-glucuronide binds to the active site via hydrogen bonds and π–type bonds (115Trp, 155Tyr, 163Asn, 144His, 112Asn, 141Glu, and 223His).

Furthermore, in the case of lipase A from *P. aeruginosa*, best binding affinity to the active site of the enzyme had luteolin 7-*O*-(2″-glucuronyl)-glucuronide, with the binding energy of −8.5 kcal/mol. This molecule also had better binding energy compared to the control molecule binding energy of −6.1 kacl/mol. Only five amino acids and their residues play roles in binding this molecule: 118Leu, 112Ser, 82Ser, 214Phe, and 16Met.

These molecular modeling studies show the high inhibitory potential of two luteolin glycosides, 7-*O*-(2″-glucuronyl)-glucuronide and luteolin-7-*O*-glucuronide, regarding their relatively high abundance in analyzed extracts compared to other flavonoid-*O*-glycosides. In the cases of in vitro testing of these extracts against the four enzymes, all tested extracts exhibited similar inhibitory potential. These results should suggest that these two compounds could be the carriers of biological activity among all compounds in the extracts.

Further studies should focus on isolating these compounds from the *V. officinalis* that showed great inhibitory potential in in silico studies and testing them as the potential carriers of biological activity, regarding their high relative abundance, and testing these compounds for the inhibitory activity of presented enzymes in vitro.

## 3. Materials and Methods

### 3.1. Plant Material and Extractions

The Institute for Medicinal Plants Research “Dr. Josif Pancic,” located in Belgrade, Serbia, provided samples of the plant material. For future usage, the aerial parts of *Verbena officinalis* L. (Verbenaceae) plant was used and processed into a fine powder. Four distinct extraction solvents, each denoted by a unique sample code, were used in this investigation. To extract the sample with the B1 label, 100% butanol was used to extract the sample with the B1 label. Butanol and ethanol mixes were used in different ratios for samples BE7.5, BE5, and BE2.5. BE7.5 used a 75% butanol to 25% ethanol mixture; BE5 used an equal mixture of 50% butanol and 50% ethanol; and BE2.5 used a 25% butanol to 75% ethanol combination. These solvents were chosen in order to evaluate the effects of varying butanol and ethanol amounts on phytochemical extraction. A 2 g sample was extracted overnight at 4 °C using 60 mL of each solvent. The following day, the samples were sonicated for 15 min and filtered through Whatman No. 4 paper. The plant residue was then re-extracted with an additional 60 mL of solvent at 4 °C over a period of 48 h, and this procedure was repeated. The extracts were subsequently evaporated to dryness at 40 °C using a rotary vacuum evaporator (Büchi R-210, Flawil, Switzerland).

### 3.2. Chemical Composition of Extracts

Using conventional colorimetric assays, the extracts total phenolic and flavonoid contents were ascertained [22]. The total phenolic content (TPC) of the extracts was determined using a modified Folin–Ciocalteu method. Briefly, 250 µL of each extract solution was mixed with 1000 µL of diluted Folin–Ciocalteu reagent (1:9, *v*/*v*) and vortexed. After 3 min, 750 µL of 1% Na_2_CO_3_ solution was added. The mixture was incubated at room temperature for 2 h, and the absorbance was recorded at 760 nm. TPC was calculated from a gallic acid calibration curve and expressed as milligrams of gallic acid equivalents per gram of extract (mg GAE/g extract). The total flavonoid content (TFC) was measured using the aluminum chloride colorimetric assay. Equal volumes (1000 µL) of extract solution and 2% AlCl_3_ in methanol were mixed and incubated for 10 min at room temperature. A blank was prepared under the same conditions, excluding AlCl_3_. Absorbance was measured at 415 nm, and the value of the blank was subtracted from that of the sample. TFC was determined using a rutin standard curve and expressed as milligrams of rutin equivalents per gram of extract (mg RE/g extract).

The metabolic profiling of the extract was performed using LC-HRMS/MS with a Thermo Scientific Vanquish Core HPLC system coupled to an Orbitrap Exploris 120 mass spectrometer. Separation was achieved on a Hypersil GOLD C18 column (50 × 2.1 mm, 1.9 μm) with a flow rate of 300 μL/min and an injection volume of 5 μL. The mobile phases consisted of ultrapure water + 0.1% formic acid (A) and acetonitrile + 0.1% formic acid (B), with a gradient elution from 5% to 95% B over 10 min, followed by re-equilibration. Detection was carried out in negative ESI mode, with full MS scans (100–1500 *m*/*z*) acquired at 60,000 resolution, and MS/MS spectra collected in data-dependent acquisition mode at 15,000 resolution using CID at 35% collision energy. Identification of phenolic compounds was based on chromatographic behavior, MS and MS^2^ fragmentation patterns, comparison with reference standards, and literature data about *Verbena* species [6,13,17,18,19,23]. Full-scan MS analysis was employed to detect the monoisotopic mass of unknown compounds, through which molecular formulas of compounds of interest were obtained. Fragmentation pathway obtained by MS^2^ fragmentation in high resolution was utilized for tentative identification of the chemical structures of the compounds. The data were processed using Xcalibur^®^ data system (version 2.1, Thermo Finnigan, Waltham, MA, USA) [24].

Using GraphPad Prism 9, heatmaps showing the distribution of grouped compounds in various extracts were created using the normalized peak area values derived from LC-HRMS/MS analysis. The relationship between the total phenolics/flavonoids and antioxidant activities was assessed by calculating the Pearson correlation coefficient and the analysis was performed by using GraphPad Prism 9.

### 3.3. Antioxidant Assays

Through the use of reducing power (FRAP and CUPRAC), radical scavenging (DPPH and ABTS), and the phosphomolybdenum assay, the antioxidant capability of each extract was evaluated [25]. For the DPPH (2,2-diphenyl-1-picrylhydrazyl) radical scavenging assay, 1 mL of each extract solution was mixed with 4 mL of a 0.004% methanolic DPPH solution. The mixture was incubated in the dark at room temperature for 30 min, after which the absorbance was measured at 517 nm. Results were expressed as milligrams of Trolox equivalents per gram of extract (mg TE/g extract). In the ABTS [2,2′-azino-bis(3-ethylbenzothiazoline-6-sulfonic acid)] assay, the ABTS^+^ radical cation was generated by reacting 7 mM ABTS with 2.45 mM potassium persulfate, followed by incubation in the dark at room temperature for 12–16 h. The resulting solution was diluted with methanol to obtain an absorbance of 0.700 ± 0.02 at 734 nm. Then, 2 mL of this working solution was mixed with the extract and incubated for 30 min at room temperature. Absorbance was measured at 734 nm, and antioxidant activity was expressed as mg TE/g extract. For the CUPRAC (cupric ion-reducing antioxidant capacity) assay, the extract solution was added to a reaction mixture containing 1 mL of 10 mM CuCl_2_, 1 mL of 7.5 mM neocuproine, and 1 mL of 1 M ammonium acetate buffer (pH 7.0). A blank was prepared under the same conditions, excluding CuCl_2_. After 30 min of incubation at room temperature, absorbance was recorded at 450 nm. Results were expressed as mg TE/g extract. In the FRAP (ferric-reducing antioxidant power) assay, 2 mL of freshly prepared FRAP reagent—comprising 0.3 M acetate buffer (pH 3.6), 10 mM TPTZ in 40 mM HCl, and 20 mM FeCl_3_ in a 10:1:1 ratio—was mixed with the extract solution. After 30 min of incubation at room temperature, absorbance was measured at 593 nm. Results were presented as mg TE/g extract. For the phosphomolybdenum assay, the extract was combined with 3 mL of reagent solution containing 0.6 M sulfuric acid, 28 mM sodium phosphate, and 4 mM ammonium molybdate. The reaction mixture was incubated at 95 °C for 90 min, and absorbance was measured at 695 nm. The total antioxidant capacity was expressed as millimoles of Trolox equivalents per gram of extract (mmol TE/g extract). In the metal-chelating activity assay, the extract was added to 0.05 mL of 2 mM FeCl_2_, followed by the addition of 0.2 mL of 5 mM ferrozine to initiate the reaction. A blank was prepared by replacing ferrozine with distilled water. After 10 min of incubation at room temperature, absorbance was measured at 562 nm. Metal-chelating activity was expressed as milligrams of EDTA equivalents per gram of extract (mg EDTAE/g extract).

### 3.4. Enzyme Inhibition Studies

The ability of each extract to inhibit the enzymes acetylcholinesterase (AChE), butyrylcholinesterase (BChE), tyrosinase, and amylase was determined using in vitro standard methods as previously reported [26,27].

### 3.5. Activity of Extracts Against Pseudomonas aeruginosa PAO1

**Figure 16:**
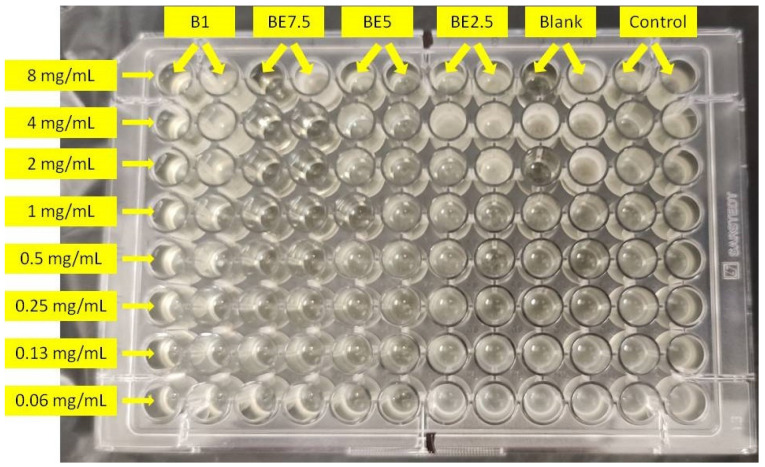
Microdilution assay setup for evaluating the antimicrobial activity of plant extracts (B1, BE7.5, BE5, BE2.5) against *Pseudomonas aeruginosa*. Serial two-fold dilutions ranging from 8 mg/mL to 0.06 mg/mL were prepared in a 96-well microtiter plate. Wells labeled ‘Blank’ contain broth only, while ‘Control’ wells contain bacterial inoculum without extract treatment.

The bacterium used in the assay was *Pseudomonas aeruginosa* PAO1 strain. The antibacterial activity of *V. officinalis* extracts were evaluated using the microdilution method in 96-well microtiter plates. Minimum inhibitory concentrations (MIC) and minimum bactericidal concentrations (MBC) of the extracts against the bacterial and yeast species were determined as previously described [28]. Ampicillin served as positive control. The experimental setup is visually presented in Figure 16

### 3.6. In Silico Study

Acetylcholinesterase (AChE, PDB ID: 7E3H), butyrylcholinesterase (BChE, PDB ID: 7AIY), pancreatic alpha amylase (PDB ID: 4GQR), tyrosinase (PDB ID: 3NQ1), elastase B (PDB ID: 8CC4), and lipase A (PDB ID: 1EX9) were target enzymes whose crystal structures were sourced from the Protein Data Bank (https://www.rcsb.org/, accessed on 1 April 2025). The enzyme that was from *Priestia megaterium* was tyrosinase; all the others were human, except elastase B and lipase A, which are from *P. aeruginosa*. The specific protein–ligand complexes’ interactions were also investigated in detail.

Tested compounds were generated first in Data Warrior [29], generating .sdf files from the Isomeric SMILES, and then the .sdf files were converted in .pdbqt format in OpenBabel GUI [30]. Preparation of tested enzymes and docking grid files were generated using AutoDockTools 1.5.7 [31], while docking was performed using AutoDock Vina [32]. For studying the protein–ligand interactions, Biovia DS Visualizer 4.5 (Dassault Systèmes Biovia Software Inc., Wien, Austria) was used. The binding energy of each ligand was calculated, and the ligands with lowest binding energy had the best binding affinity to the enzyme active site. The active site of each enzyme was determined based on the co-crystalized control molecule within the enzyme.

## 4. Conclusions

The present study highlights the rich phytochemical profile and promising bioactivity of common vervain extracts obtained using various butanol-ethanol solvent systems. A total of 75 compounds spanning multiple phytochemical classes—including phenolic acids, flavonoids, iridoids, phenylethanoids, and xanthones—were identified through LC-HRMS/MS analysis. Heatmap visualization revealed solvent-dependent extraction efficiencies, with BE5 and BE2.5 emerging as the optimal system for the recovery of key antioxidant and enzyme-inhibiting compounds, such as verbascoside, luteolin derivatives, and verbenalin.

Biological assays confirmed BE5 as the extract with the highest antioxidant potential across DPPH, ABTS, CUPRAC, FRAP, and PMA assays, while BE2.5 showed superior metal-chelating and antibacterial activity against *Pseudomonas aeruginosa*. In silico docking studies further validated the bioactivity of the most abundant compounds, with luteolin 7-*O*-(2″-glucuronyl)-glucuronide consistently demonstrating the highest binding affinity across five tested enzymes, including those relevant to metabolic and neurodegenerative disorders and bacterial virulence., while verbasoside was the best match as a tyrosinase inhibitor.

These findings support the potential of *V. officinalis* as a valuable source of antioxidant and enzyme-inhibiting agents. While antibacterial activity was confirmed only against a single bacterial species, the results provide a strong rationale for further investigation into its broader antimicrobial spectrum. The study also underscores the importance of solvent optimization in maximizing phytochemical yield and biological performance. The consistent demonstration of antioxidant, antimicrobial, and multitarget enzyme-inhibitory properties, supported by in silico molecular modeling, highlights the genus’ potential for the development of therapeutic agents targeting oxidative stress, microbial virulence, and enzyme-regulated pathologies. Together, these findings offer a comprehensive foundation for future drug discovery and functional product development from *Verbena* species.

## Figures and Tables

**Table 1:** Total phenolics (TP) and total flavonoids (TF) content in common vervain extracts.

Sample	TP (mg GAE/g)	TF (mg RE/g)
B 1	29.04 ± 0.01	6.90 ± 0.07
BE 7.5	25.31 ± 0.15	6.16 ± 0.04
BE 5	69.55 ± 1.07	15.33 ± 0.20
BE 2.5	30.06 ± 0.22	8.45 ± 0.06

GAE—galic acid equivalents, RE—rutin equivalents.

**Table 2:** Antioxidative activities of *Verbena officinalis* extracts.

Sample	DPPH (mg TE/g)	ABTS (mg TE/g)	CUPRAC (mg TE/g)	FRAP (mg TE/g)	MCA (mg EDTA/g)	PMA (mmol TE/g)
B 1	66.54 ± 0.63	66.91 ± 1.23	131.14 ± 1.57	80.11 ± 0.33	18.55 ± 1.13	1.66 ± 0.04
BE 7.5	80.85 ± 0.43	83.01 ± 1.72	141.36 ± 4.02	89.73 ± 0.23	17.25 ± 0.39	1.72 ± 0.03
BE 5	181.03 ± 0.13	178.50 ± 2.10	287.86 ± 4.51	170.99 ± 1.98	10.55 ± 0.24	3.62 ± 0.08
BE 2.5	57.91 ± 0.38	81.76 ± 1.68	88.71 ±0.68	65.25 ± 1.59	21.83 ± 0.27	3.62 ± 0.01

DPPH-1,1-diphenyl-2-picrylhydrazil assay; TE—Trolox equivalents; ABTS-2,2′-azino-bis(3-ethylbenzothiazoline-6-sulphonate) assay; CUPRAC—Cupric ion-reducing antioxidant capacity assay; FRAP—ferric-reducing/antioxidant power; EDTA—ethylenediaminetetraacetic acid equivalents; MCA—metal-chelating assay; PMA—phosphomolybdenum assay.

**Table 3:** Enzyme inhibitory effects of *Verbena officinalis* extracts.

Sample	AChE Inhibition (mg GALAE/g)	BChE Inhibition (mg GALAE/g)	Tyrosinase Inhibition (mg KAE/g)	Amylase Inhibition (ACAE/g)
B 1	5.11 ± 0.01	12.82 ± 0.03	91.91 ± 1.13	0.48 ± 0.00
BE 7.5	5.17 ± 0.02	12.53 ± 0.05	94.75 ± 0.64	0.48 ± 0.00
BE 5	5.10 ± 0.02	11.31 ± 0.14	102.33 ± 1.03	0.59 ± 0.01
BE 2.5	5.13 ± 0.01	11.95 ± 0.08	95.76 ± 0.97	0.29 ± 0.04

GALAE—galantamine equivalents; KAE—kojic acid equivalents; ACAE—acarbose equivalents.

**Table 4:** Antimicrobial activity of extracts against *Pseudomonas aeruginosa* (mg/mL).

	B1		BE7.5		BE5		BE2.5		Ampicillin	
Bacterium	MIC	MBC	MIC	MBC	MIC	MBC	MIC	MBC	MIC	MBC
*P. aeruginosa*	1.00	2.00	1.00	2.00	0.50	1.00	0.25	0.50	0.003	0.005

**Table 5:** Docking score values of the compounds to acetylcholinesterase.

Compounds	Affinity (kcal/mol)
Hastatoside	−7.6
Luteolin 7-*O*-(2″-glucuronyl)-glucuronide	−10.1
Luteolin-7-*O*-glucuronide	−9.5
Martynoside	−9.7
Verbascoside	−9.6
Verbenalin	−7.9
1-benzyl-4-[(5,6-dimethoxy-1-indanon-2-yl)methyl]piperidine	−11.4

**Table 6:** Docking score values of the compounds to butyrylcholinesterase.

Compounds	Affinity (kcal/mol)
Hastatoside	−8.4
Luteolin 7-*O*-(2″-glucuronyl)-glucuronide	−10.3
Luteolin-7-*O*-glucuronide	−10.1
Martynoside	−9.6
Verbascoside	−9.9
Verbenalin (kcal/mol)	−8.3
2-{1-[4-(12-Amino-3-chloro-6,7,10,11-tetrahydro-7,11-methanocycloocta[b]quinolin-9-yl)butyl]-1*H*-1,2,3-triazol-4-yl}-*N*-[4-hydroxy-3-methoxybenzyl]acetamide	−10.7

**Table 7:** Docking score values of the compounds to α-amylase.

Compounds	Affinity (kcal/mol)
Hastatoside	−7.4
Luteolin 7-*O*-(2″-glucuronyl)-glucuronide	−9.5
Luteolin-7-*O*-glucuronide	−9.4
Martynoside	−8.5
Verbascoside	−8.5
Verbenalin	−7.5
3,5,7-trihydroxy-2-(3,4,5-trihydroxyphenyl)-4*H*-chromen-4-one	−7.8

**Table 8:** Docking score values of the compounds to tyrosinase.

Compounds	Affinity (kcal/mol)
Hastatoside	−6.3
Luteolin 7-*O*-(2″-glucuronyl)-glucuronide	−7.5
Luteolin-7-*O*-glucuronide	−7.9
Martynoside	−6.9
Verbascoside	−7.7
Verbenalin	−6.9
Kojic acid	−5.4

**Table 9:** Docking score values of the compounds to elastase B.

Compounds	Affinity (kcal/mol)
Hastatoside	−7.1
Luteolin 7-*O*-(2″-glucuronyl)-glucuronide	−9.6
Luteolin-7-*O*-glucuronide	−9.1
Martynoside	−8.8
Verbascoside	−8.6
Verbenalin	−7.4
Inhibitor (control) *	−7.6

* [(2~{R})-4-methyl-1-oxidanylidene-1-[[4-(trifluoromethyl)phenyl]amino]pentan-2 yl]phosphonic acid.

**Table 10:** Docking score values of the compounds to lipase A.

Compounds	Affinity (kcal/mol)
Hastatoside	−6.6
Luteolin 7-*O*-(2″-glucuronyl)-glucuronide	−8.5
Luteolin-7-*O*-glucuronide	−8.1
Martynoside	−7.8
Verbascoside	−8.2
Verbenalin	−6.8
Inhibitor (control) *	−6.1

* octyl-phosphinic acid 1,2-bis-octylcarbamoyloxy-ethyl ester.

## Data Availability

The original contributions presented in this study are included in the article/Supplementary Materials. Further inquiries can be directed to the corresponding authors.

## Supplementary Materials

The following supporting information can be downloaded at: https://www.mdpi.com/article/10.3390/ph18071012/s1, Table S1: The compounds found in the extracts of *V. officinalis* identified by their names, retention times (*t*_R_, min), molecular formulas, MS data (calculated and exact masses, as well as mean mass accuracy—Δ ppm), and significant MS^2^ fragment ions; Figure S1: Base peak chromatograms of all four different extracts.
