# Supplementary figures and images for: Investigation of Bioactive Compounds Extracted from Verbena officinalis and Their Biological Effects in the Extraction by Four Butanol/Ethanol Solvent Combinations
Source: Pharmaceuticals (Basel). 2025 Jul 7;18(7):1012. doi: 10.3390/ph18071012 (PMC12300994; doi:10.3390/ph18071012)

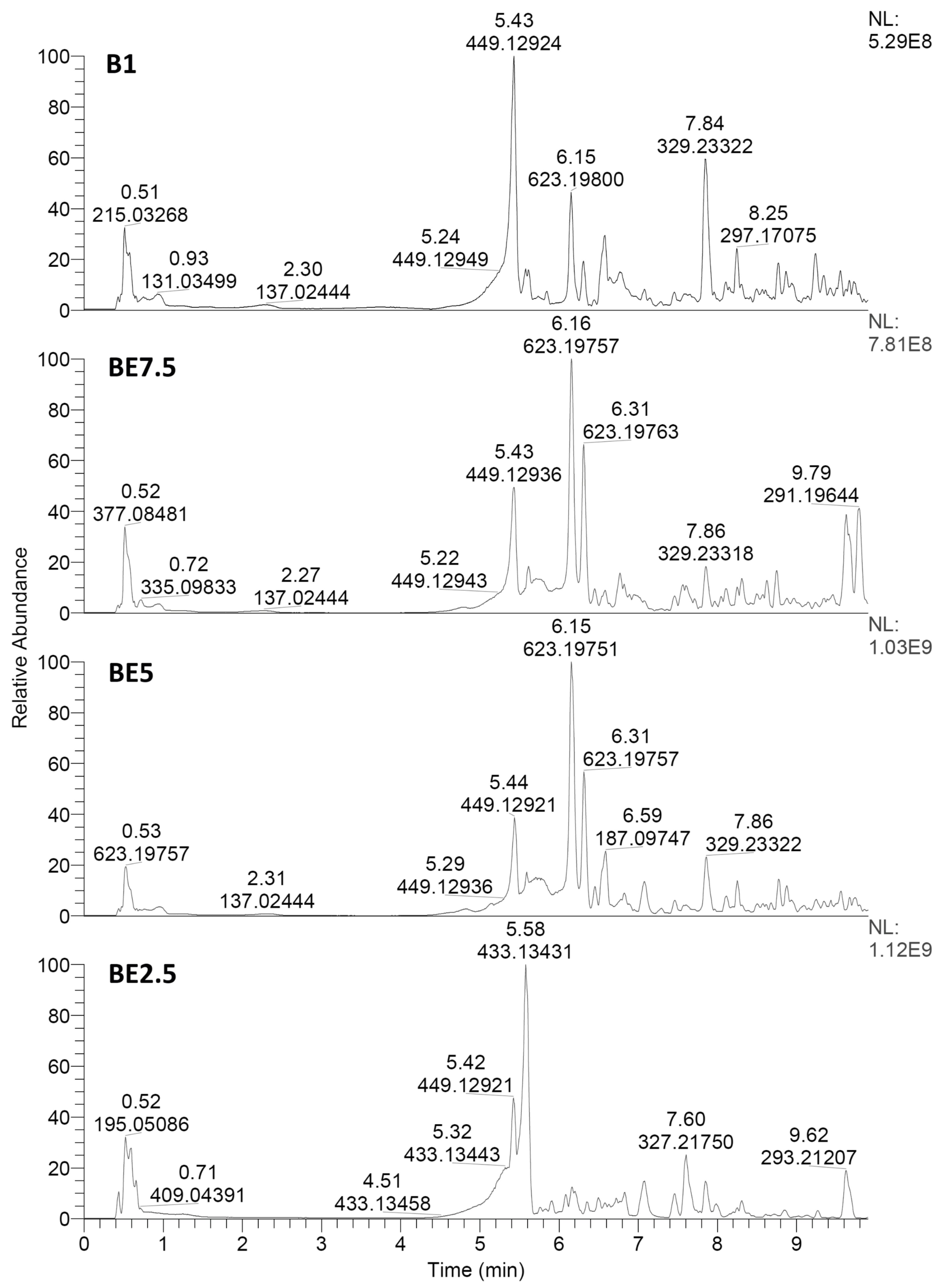

Supplement: Supplementary file 1 [file pharmaceuticals-18-01012-s001.zip › Figure S1.tif]
